# Nurse educators' knowledge and opinions about the “One Health” approach

**DOI:** 10.1111/inr.12983

**Published:** 2024-05-07

**Authors:** Nilay Ercan Şahin, Mücahide Öner

**Affiliations:** ^1^ Nursing Faculty Hacettepe University Ankara Turkey; ^2^ Nursing Department Bitlis Eren University, Health Science Faculty Bitlis Turkey

**Keywords:** Nurse educators, nursing education, One Health

## Abstract

**Aim:**

This study was conducted to reveal the knowledge and opinions of Turkish nurse educators about the One Health approach.

**Background:**

One Health is a collaborative, multisectoral, and transdisciplinary approach working at local, regional, national, and global levels to achieve optimal health (and well‐being) outcomes recognizing the interconnections between people, animals, plants, and their shared environment. Despite nurses' unique position to address inequities in health care for all people around the world, the concept of One Health is a relatively new concept in nursing.

**Methods:**

This is a cross‐sectional descriptive study involving a total of 272 nurse educators from various universities in Turkey. The email addresses of nurse educators were obtained from university websites, and the questionnaire form (created using the survey tool Google Forms) was then sent to them. Open‐ended questions underwent content analysis, while multiple‐choice questions were analyzed in terms of numbers and percentages.

**Results:**

Eighty‐two percent of nurse educators had never heard of or encountered the One Health approach before. Ninety‐six percent of nurse educators think that the One Health approach should be included in nursing education. Nurse educators asserted that nursing should be included as a discipline in the One Health approach due to its relationship with the environment, health prevention and promotion, and its close contact with society.

**Conclusion:**

The results of this study underscore the necessity of enhancing One Health knowledge among nursing educators. It is particularly significant that the majority of nurse educators had not previously encountered or been aware of the One Health approach, highlighting an important gap in awareness and understanding.

**Implications for nursing and nursing policy:**

Nursing, silent in the realm of One Health, should integrate this approach, which encompasses human, animal, and environmental health, into nursing education, research, and practice. It is time for action to incorporate One Health into both undergraduate and graduate nursing education programs, conducting research in this area, and fostering collaborations.

## INTRODUCTION

Over the past 20 years, events such as the swine flu, SARS, MERS, Zika, Ebola, and most recently, the COVID‐19 pandemic have underscored the critical need for collaboration between the veterinary and human health sectors. These occurrences have heightened awareness regarding the importance of joint efforts to prevent and control zoonotic infections and antibiotic resistance (Sikkema & Koopmans, 2016). Furthermore, given the escalating global population and urbanization trends, it is evident that the philosophy of “One World, One Health” must be embraced from the global level down to the most local level. This approach is essential to mitigate the risk of pandemic outbreaks, which are anticipated to occur with greater frequency (Jorwal et al., 2020). In this context, the concept of “One Health,” which extends beyond traditional healthcare boundaries, plays a pivotal role in achieving improved public health outcomes. Given the increasingly complex conditions facing humanity in current times, embracing this broader concept is essential for addressing interconnected health challenges effectively (Jorwal et al., 2020).

According to the One Health Commission, “One Health is a collaborative, multisectoral, and transdisciplinary approach—working at local, regional, national, and global levels—to achieve optimal health (and well‐being) outcomes recognizing the interconnections between people, animals, plants, and their shared environment.”(What is One Health? 2023).

The concept of “One Health” is not a novel idea. Its roots trace back to the writings of Hippocrates, who highlighted the connection between a clean environment and public health, underscoring the enduring relevance of this holistic approach throughout history. (Yılmaz et al., 2018). In the 1960s, it was claimed that there was no paradigm difference between human and animal medicine, and the concept of “One Medicine” was put forward for the first time (Atlas, 2013). The concept put forward by Schwabe as “One Medicine” changed in the first years of the 21st century and began to be considered as “One Health” (Kaplan & Scott, 2011). Initially, the concept of “One Health” emerged as a framework for fostering collaboration and finding solutions in combating infectious diseases, particularly zoonotic diseases. As time progressed, additional issues such as antimicrobial resistance, food safety, environmental health, laboratory services, and neglected tropical diseases have been incorporated into the scope of concerns addressed by the One Health approach (One Health, 2023).

As per the One Health definition, it is a collaborative, multisectoral, and transdisciplinary approach. It includes ecology, zoology, botany, agriculture, social science,  urban planning, engineering, and natural resource management, as well as health fields such as veterinary and medicine. It is a discipline that should be included in the One Health approach in nursing.

Despite nurses' unique position to address inequities in health care for all people around the world, the concept of One Health is a relatively new concept in nursing (Premji & Hatfield, 2016). The theory of Florence Nightingale, the founder of modern nursing, which emphasizes the importance of protecting and maintaining the relationship between human health and environmental health, actually shows how close the nursing profession is to the One Health approach (Lee et al., 2013). Moreover, The American Nursing Association endorsed One Health in 2010 with the following statement: “Nurses recognize all forms of life on Earth are interconnected and interdependent. The One Health Initiative epitomizes this fundamental concept. The American Nurses Association is proud to be a supporter of this ground‐breaking initiative. Working together, we will enhance and improve the health of humans, animals and the environment” (Redican & Akpinar‐Elci, 2021). However, upon reviewing the literature, it becomes evident that nurses have remained relatively silent about the One Health approach for a considerable period of time. Therefore, it is imperative for the nursing profession to give greater attention to the issue of One Health. In the nursing literature, there are several studies examining the relationship between environmental pollution and human health (Nahidi et al., 2013; Puett et al., 2014; Riha et al., 2014). However, there are no comprehensive studies on the One Health approach.

Additionally, it has been observed that One Health is included in postgraduate programs in medicine and public health, primarily in veterinary medicine. Although it is not seen as a course in nursing education, it is not possible to make an opinion about whether it is included in existing courses. In the literature, it is suggested that One Health education should commence early in the undergraduate years and draw upon various teaching traditions and knowledge systems to establish an effective reinforcing cycle of transdisciplinary thinking and holistic approaches. This approach is asserted to maximize the inclusiveness and diversity of student groups and promote holistic conceptions of One Health beyond pathogen‐centric science. (Villanueva‐Cabezas et al., 2022).

Through holistic care practices, nurses evaluate the individual as a whole, recognizing the interconnectedness of body, mind, soul, emotions, environment, relationships, and the social and cultural dimensions of life (Bayındır, 2019). Therefore, nurses have a critical role in reducing exposure to these environmental risks, and problems such as reemerging zoonotic diseases, antimicrobial resistance, climate change, food safety, and environmental pollution are important threats to human health. Numerous instances exist of nurses leading these. For example, nurses also fulfill their important roles and responsibilities in the prevention, care, and protection of infectious diseases. With the COVID‐19 pandemic, the visibility of nurses has increased like never before, and the importance of nursing care in the fight against infectious diseases has been understood (ICN, 2021). Moreover, nurses have been at the forefront of addressing the challenges posed by climate change and advocating for actions to mitigate health impacts at both individual and societal levels. Nurses were honored with the award for their exemplary work in climate change and public health in America (Anderko et al., 2017). Premji and Hatfield argue that for the nursing profession to play a significant role in advancing the objectives of the sustainable development goals, nurses must perceive themselves as collaborators with their international counterparts, engaging in knowledge cocreation and exchanging ideas and best practices to explore innovative solutions for common health challenges (Premji & Hatfield, 2016).

The One Health approach provides a framework of core competencies that include areas such as management, communication and informatics, values and ethics, leadership, team and collaboration, roles and responsibilities, and systems thinking. These shared competencies are particularly important in guiding health professions curricula in a world where zoonotic diseases are emerging, interactions between humans and animals are increasing, and the ongoing impacts of climate change are present (Larsen, 2021). Upon reviewing the literature on One Health, studies within the fields of public health, human medicine, and veterinary medicine were identified, focusing on core competencies, curriculum development, and evaluation, as well as students' perceptions of One Health education (Chakraborty et al., 2022; Larsen, 2021; Shrivastava & Shrivastava, 2022; Togami et al., 2018). Accordingly, defining, developing, evaluating, improving, and continually refining One Health education are crucial within nursing curricula. However, no studies pertaining to One Health education in the nursing field have been identified. In this context, the present study was designed to reveal nurse educators regarding their knowledge of the One Health approach and its integration into nursing education. It is anticipated that this study will aid in advancing further research and educational strategies in One Health, a multidisciplinary approach, by uncovering nurse educators' understanding of One Health and their opinions on its incorporation into nursing education.

For this purpose, answers to the following questions were sought in this study:

1. What is the nurse educators' knowledge about the One Health approach?
2. If nurse educators have knowledge about the One Health approach, what is their opinion regarding its integration into nursing education?

## METHODS

### Study design

This study was conducted as a cross‐sectional descriptive study, using multiple‐choice and open‐ended questions.

### Participants and sampling

The population of the research comprised nurse educators who are part of the academic staff at 47 private and 105 state universities in Turkey. They were invited to participate in the study via email (*N*  =  2,086). The nurse educators who agreed to participate in the present study comprised the study sample (*n*  =  272). Table 1 shows the average age, working experience, and titles of the participants.

**TABLE 1:** Distribution of participants’ age, work period, and title.

Characteristics	Mean + SD	
Age	37.8 4 + 8.91 (min: 23 max: 64)	
	*n*	%
Gender		
Female	236	86.76
Male	36	13.24
Working experience		
0–10 years	131	48.16
11–20 years	69	25.36
21 or more years	72	26.48
Title		
Lecturer	37	13.60
Research assistant	89	32.72
Assistant professor	87	31.98
Associate professor	36	13.23
Professor	23	8.47
**Total**	**272**	**100**

### Data collection tool

The questionnaire was developed based on a literature review conducted by the researchers. The survey form included multiple‐choice questions regarding participants' age, work experience, and title. It also asked whether they had previously heard of One Health, where they first encountered it, whether it should be incorporated into nursing education, at what level of education it should be included, and how it should be integrated into nursing education.

Furthermore, open‐ended questions were utilized to assess the definition of One Health, the involved disciplines, and the topics it encompasses. The questionnaire underwent content validity evaluation by five experts, whose opinions were solicited. Subsequent adjustments to the form were made based on their recommendations. All content validity index scores surpassed 0.82. Following the expert opinions, a small pilot study involving 10 nursing educators who volunteered to participate was conducted. During the pilot study, nursing educators encountered no difficulties with the questionnaires. Consequently, no changes were made to the questionnaire form as a result of the pilot study.

While the survey ended for participants who had never heard of the One Health approach, those who knew about One Health were directed to subsequent questions.

### Data collection

The aim was to reach all nurse educators in undergraduate nursing education in Turkey. To achieve this, the email addresses of nurse educators were obtained from the websites of 140 universities, and the questionnaire form (created using the survey tool Google Forms) link was sent to them. The questionnaire form was initially sent via email on July 7, 2023. Subsequently, reminder emails were sent three times until September 20, 2023. The study concluded with the participation of nurse educators (*n* = 272) who completed the questionnaire and agreed to participate in the survey.

### Data analyses

Open‐ended questions were analyzed using content analysis, and multiple‐choice questions were analyzed as numbers and percentages.

### Ethical considerations

This study was approved by the Ethics Board of University (permission no: E‐.3852, date: 03.07.2023). In addition, participants completed the questionnaire after agreeing to complete the survey through the link provided. Participants provided electronic informed consent. The study was conducted in accordance with the Helsinki principles.

## RESULTS

In this study, which was conducted to reveal the knowledge and thoughts of nurse educators about the One Health approach, multiple‐choice questions were demonstrated with numbers and percentages. Open‐ended questions were grouped by content analysis, and examples of participant answers along with numbers and percentages are included in the tables.

Table 2 shows whether the participants have heard of One Health before, where they first heard about it, and their thoughts about taking part in nursing and nurse education. Nurse educators were asked about the disciplines included in the One Health approach. The first three places were medicine, veterinary medicine, and nursing. Only 21 of the 51 nurse educators who had knowledge about One Health stated that nursing is included as a discipline in the One Health approach.

**TABLE 2:** Nurse educators' knowledge and opinions about the One Health approach.

**Do you know the One Health approach?**		
Yes	51	18.80
No	221	81.20
**Where did you first hear or encounter it?**		
During postgraduate education	19	37.25
While reading scientific articles	21	41.17
At the scientific congress	7	13.73
Others^a^	4	7.85
**What are the disciplines involved in One Health?** ^b^		
Medicine	31	60.78
Veterinary	24	47.05
Nursing	21	41.17
All disciplines	10	19.60
Biology	10	19.60
Agriculture engineering	8	15.68
Zoology	7	13.72
Environmental engineering	7	13.72
Pharmaceuticals	5	9.80
Policy maker	5	9.80
**Should the One Health approach be included in nursing education?** ^c^		
Yes	49	96.07
No	2	3.93
**Which level of education should One Health be given?** ^b^		
Undergraduate level	45	88.23
Master level	36	70.58
PhD. level	28	54.90
**How should the One Health approach be taught as a course?**		
Within the scope of nursing basic vocational courses	22	43.13
As a compulsory course	15	29.41
As an elective course	14	27.46
**In which branch of nursing do you think the One Health approach should be included?** ^b^		
Public Health Nursing	47	94
Fundamentals of Nursing	20	40
Internal and Surgical Nursing	17	34
Pediatric Nursing	12	24
Nursing Management	10	20
Obstetric Nursing	8	16
Mental Health Nursing	7	14

^a^ Social media and scientific meeting.

^b^ Participants marked more than one option. It was used the row percents.

^c^ Those who know the One Health approach answered.

Nurse educators who said they knew One Health were asked to define One Health. Content analysis was made in the definitions, and groups were given as numbers and percentages based on the themes. Additionally, a few examples of definitions made by nurse educators are given (Table 3).

**TABLE 3:** Nurse educators’ definition of One Health.

Themes	*n*	%	Quotes
A multidisciplinary and collaborative concept	17	33.33	“It is a multidisciplinary and collaborative concept that takes into account all factors affecting health, where many professional groups work together to protect and improve human health.” (P8) “All disciplines working together for global health.” (P21) “It is an approach that includes interdisciplinary collaboration on the life and health of all living things, including humans, animals and the environment, at international and national levels.” (P94) “It is all disciplines working together for global health.” (P157)
A whole	13	25.50	“It is an approach to consider environmental health, human health and animal health as a whole.” (P46) “It is the evaluation of humans, animals and the environment as a whole in order to maintain the continuity of well‐being.” (P77) “An approach that considers the health of all living things and the environment as a whole.” (P205)
Single roof	7	13.72	“It is a concept that combines environment, human and animal health under single roof.” (P227) “It is the gathering of human, animal and environmental health under single roof.” (P15) “For the health; It is the gathering of human, zoonotic and environmental health under single roof.” (P183)
Communicable disease management	7	13.72	“It is an approach against zoonotic diseases.” (P31) “In order to fight against infectious diseases, the whole environment, humans and animals, which can be effective, are taken together and appropriate activities are carried out.” (P67) “Fighting zoonotic diseases is a collaboration between stakeholders.” (P109)
Optimal health	3	5.88	“It is a collaborative approach to achieving optimal health.” (P11) “It is to achieve optimal health for all living and life beings in the world.” (P175) “It is to ensure optimal health for all living beings at an international level.” (P263)
Human and animal health	2	3.93	“Evaluation of human and animal health together.” (P52)
No response	2	3.92	

Table 4 shows the nurse educators’ opinion about nursing as a discipline at One Health. Participants emphasized the relationship between nursing and health, and environment, and health prevention and promotion, and close contact with the community.

**TABLE 4:** Nurse educators’ opinions on nursing as a discipline within the framework of One Health.

Theme	*n*	%	Quotes
Relation to health	23	45.11	“Since human health is at stake, nursing should be involved in every step.” (P5) “Because nursing should be included in every concept that concerns human health.” (P27) “It should be considered as a profession directly related to individual, family and public health.” (P89) “Nursing as a profession should be everywhere because it is on the front lines in the fight against diseases.” (P201)
Relation to environment	10	19.61	“One of the basic concepts that form the conceptual framework of nursing is environment. It is the nurse's responsibility to create a healthy environment by including the environment in the care process.” (P243) “Yes, I think that nursing is the closest specialty to this approach, as it is one of the rare disciplines that treats humans as a whole with their environment.” (P271)
Health prevention and promotion	9	17.64	“Nursing is a profession that takes part in the prevention and promotion of health in every place and time where the concept of human being exists, so it should definitely take part in it.” (P158) “Because of preventive health services as they play an active role, they should take part in.” (P180)
Close contact with community	4	7.84	“Nurses should be involved because they are in close contact with community.” (P64) “Because it is the professional group that comes into first and most frequent contact with the family and the individual.” (P29)

^a^ One participant thinks that nursing should not be included in One Health and four participants did not answer the question (*n* = 5; 9.8%).

## DISCUSSION

In this research aimed at revealing the knowledge and opinions of nurse educators regarding One Health, three out of four participants indicated that they had never encountered the One Health approach previously. Considering the titles of the participants, half of whom hold the titles of assistant professor, associate professor, or professor, it is notable that three‐quarters of the participants are unfamiliar with the One Health approach. Based on this result, it appears that nurse educators have no idea about the One Health approach.

The majority of nurse educators who were already familiar with the One Health approach reported encountering it initially through scientific articles or during their postgraduate education. Nurse educators who were familiar with the One Health approach were asked the question, “What is One Health?” While only two participants did not make any definition, two participants described it as an approach that addresses human and animal health. Moreover, while nurse educators point out that One Health is collaborative and multidisciplinary, some have pointed out that humans, animals, and the environment are evaluated as a whole. Furthermore, while some described One Health as a single roof, others emphasized its role in managing infectious diseases and promoting optimal health. It can be concluded that nurse educators who indicated familiarity with One Health provided definitions at a basic level.

The majority of nurse educators have expressed the importance of incorporating One Health into nursing education, citing nursing's inherent connection with health, its interface with the environment, its focus on health promotion and prevention, and its intimate engagement with society. In the literature, it is suggested that the One Health approach should not only be included in veterinary and medical education (Villanueva‐Cabezas et al., 2022) but also integrated into health professions education programs and their curricula across all levels and fields of health professional learners (Larsen, 2021).

In the present study, almost all of the nurse educators expressed the belief that the One Health approach should be integrated into undergraduate and master's level education, while more than half of the nurse educators advocated for its inclusion at the PhD level. Although not reflected in the tables, participants provided reasons for their suggested levels of education. Nurse educators highlighted that since the majority of nurses in practice hold undergraduate degrees, it is imperative to incorporate One Health into undergraduate education to enhance awareness. At the graduate level, nurse educators emphasized the necessity of integrating One Health into graduate education to facilitate comprehensive research, practice, and policy development. Villanueva‐Cabezas et al. (2022) suggested that One Health approach should be included in undergraduate programs rather than postgraduate programs. Moreover, One Health education should start at the beginning of the undergraduate years and include interdisciplinary thinking and holistic approaches. Thus, students will be encouraged to develop a holistic understanding of One Health (Villanueva‐Cabezas et al., 2020). Therefore, teaching the One Health approach should prioritize competency‐based education, engaging learners in practical training to enhance coordination and collaborative problem‐solving skills (Machalaba et al., 2018). The courses can incorporate collaborative, group‐based active learning activities, such as site visits and gallery‐based tasks, to encourage student exploration of the weekly One Health themes. This exploration can be bolstered by presentations from a diverse range of cross‐disciplinary guest experts (Villanueva‐Cabezas et al., 2022). Nursing degree programs should be constructed based on a framework of core competencies, with a particular emphasis on practical skills development (Togami et al., 2018).

In this study, most nurse educators taught that the One Health approach be included within the scope of nursing basic vocational courses. The majority of participants stated that the One Health approach should be included within the scope of public health nursing. Nurse educators may have considered including the concept of One Health in public health nursing due to its perceived alignment with public health principles. It is recommended that both public health and health education programs (e.g., community health and health promotion) are in an ideal position to integrate a One Health approach into their educational programs (Redican & Akpinar‐Elci, 2021).

### Limitations

The small sample size limits the generalizability of the findings among nurse educators globally. Therefore, there is a need for international studies with larger samples that encompass nurse educators' knowledge, opinions, and experiences regarding One Health. Additionally, conducting qualitative research with nurse educators is essential to integrate the One Health approach into nursing education curricula.

## CONCLUSION

This study aims to reveal the knowledge and opinions of Turkish nurse educators about the One Health approach. The findings underscore the necessity of enhancing One Health knowledge among nursing educators. It is particularly significant that the majority of nurse educators had not previously encountered or been aware of the One Health approach, highlighting an important gap in awareness and understanding. Among those familiar with it, nurse educators believe that the concept of One Health should be incorporated into undergraduate nursing education programs, emphasizing the importance of nursing students graduating with this awareness.

### Implications for nursing and nursing policy

For the nursing profession to effectively support the One Health approach, it must collaborate with international colleagues, cogenerate knowledge, and exchange ideas and best practices. This collaborative effort is essential for seeking innovative solutions to common health problems (Premji & Hatfield, 2016). Nurses must engage in novel ways within this social process by cultivating competencies to advance social, economic, and political action (Marmot et al., 2008). This not only involves uncovering health inequalities (e.g., social determinants of health) but also identifying innovative approaches to reform healthcare delivery (Premji & Hatfield, 2016). Nurses possess the potential and interest to engage in addressing numerous global health challenges, including noncommunicable diseases (ICN, 2011). Nurses must enhance their comprehension and discourse surrounding global health while expanding their contribution to health reform initiatives aimed at achieving health for all. (Blaney, 2012; Klopper & Hill, 2015). In this context, nursing, which has remained silent in One Health, should include this approach, which addresses human, animal, and environmental health, in nursing education, research, and practice.

It is time for action to integrate One Health into undergraduate and graduate education programs, to conduct research on One Health, and to foster collaborations. The development of competencies (political leadership, teamwork, etc.) and traits (influence, professional credibility, etc.) necessary for nurses to participate in global health and health reform must be facilitated by professional nursing education programs (Fyffe, 2009; Jivraj Shariff, 2015). It should also inquire whether current nursing curricula raise awareness about the blending boundaries between human and animal health, as well as the potential impacts of global connectedness on healthcare systems (Larsen, 2021).

## AUTHOR CONTRIBUTIONS

*Study design*: NEŞ. *Data collection*: MÖ. *Data analysis*: NEŞ. *Study supervision*: NEŞ. *Manuscript writing*: NEŞ. *Critical revisions for important intellectual content*: NEŞ, MÖ.

## CONFLICT OF INTEREST STATEMENT

The authors declared no potential conflicts of interest with respect to the research, authorship, and/or publication of this article.

## FUNDING INFORMATION

This research did not receive any specific grant from funding agencies in the public, commercial, or not‐for‐profit sectors.

## ETHICAL CONSIDERATION

This study was approved by the University of Ethical Board (Permission no: E.3852, date: 03.07.2023). Participants completed the questionnaire after agreeing to complete the survey through the link provided.
